# SET-ing the stage for PI3Kβ inhibitor sensitivity in clear cell renal cell carcinoma

**DOI:** 10.18632/oncotarget.26709

**Published:** 2019-02-22

**Authors:** Patrick G. Pilié, Eric Jonasch

**Affiliations:** Department of Genitourinary Medical Oncology, MD Anderson Cancer Center, Houston, TX, USA

**Keywords:** renal cell carcinoma, PI3K, SETD2, sensitivity, synthetic lethality

Genomic profiling of patients with clear cell renal cell carcinoma (ccRCC) has consistently shown that inactivation by mutation or methylation of the *Von Hippel Lindau* gene is a founder event in ccRCC carcinogenesis. In addition, loss of chromosome 3p-the chromosome on which *VHL* and other key drivers of ccRCC reside-is the most frequent chromosomal aberration seen in ccRCC [1]. The bi-allelic loss of *VHL* in ccRCC has multiple downstream effects, including HIF dysregulation and a subsequent pro-angiogenic state. Other 3p genes including SWI/SNF complex gene *PBRM1*, the histone deubiquitinase *BAP1*, as well as the histone methyltransferase *SETD2* are found to be mutated in 40-50%, 10-15%, and 10-15% ccRCC tumors, respectively and result in differential impact on RCC aggressiveness and prognosis [2]. In addition, ccRCC displays activation of the mTOR/PI3K/AKT axis that increases with stage, despite a relatively sparse occurrence of mutations in these genes [3].

ccRCC is notoriously resistant to traditional DNA-damaging chemotherapies, and approved treatments for metastatic disease include immune checkpoint therapy, anti-angiogenic tyrosine kinase inhibitors, and mTOR inhibition. Despite having detailed knowledge of the genomic makeup of ccRCC, treatments for advanced disease are not currently biomarker-directed or tailored to targetable mutations [4]. Furthermore, variable responses and inevitable progression plague these current approved cytostatic targeted therapies for treating ccRCC. Thus, preclinical and clinical studies to inform biomarker development and guide treatment selection and sequence are greatly needed.

In this current study, Terzo and Lim *et al* present preclinical data showing that *SETD2* mutant RCC cells and xenograft RCC tumors display increased sensitivity, as evidenced by decreased cell viability and cellular proliferation, to PI3Kβ-specific inhibition compared to *SETD2* wildtype cells. Furthermore, PI3Kβ and pan-PI3K, but not PI3Kα, inhibition leads to reduced cell viability and migration in a *SETD2*-loss dependent manner. In addition, the AKT-specific inhibitor MK2206 led to similar anti-cancer activity and reduced pS6 phosphorylation levels only in *SETD2*-deficient cells, suggesting that AKT is a key effector linking SETD2 to PI3Kβ. Lastly, the authors show promising *in vivo* efficacy data for the use of AZD8186, a PI3Kβ-specific inhibitor, in *SETD2*-deficient A498 xenografts versus *SETD2*-proficient 786-O xenografts.

Clinical studies with PI3K or AKT inhibitors in patients with ccRCC have shown mixed results. For example, a phase 2 study of the AKT-inhibitor MK2206 in second-line setting for patients with metastatic ccRCC did not meet its primary efficacy endpoint and showed a high incidence of adverse events [5, 6]. However, in contrast to disease-stability seen with the everolimus arm, there was a small number of patients that achieved partial and even one complete response in the MK2206 arm [5, 6]. Genomic analysis did not reveal any association between response and 3p gene mutations, but sample size was small and analyses were performed on primary, as opposed to metastatic tumors, possibly missing key driver gene alterations [5]. Clinical studies of pan-PI3K inhibitors have shown similar mixed-results; however, it is important to note that no clinical study thus far has been performed in a biomarker-selected fashion nor has there been a clinical trial reported on PI3Kβ-specific inhibition [5].

SETD2 loss has also previously been shown to predict for sensitivity to inhibitors outside of the PI3K-pathway. Recently, preclinical studies have shown that *SETD2*-mutant cells display significantly increased sensitivity to pharmacologic inhibition of WEE1, a key modulator of the G2/M checkpoint and regulator of nucleotide resources in the cell [7]. This increased sensitivity is due to loss of H3K36me3 in *SETD2*-mutant cells and subsequent downstream depletion of nucleotide pools, with these preclinical studies spurring assessment of a first in-class WEE1 inhibitor AZD1775 in patients with *SETD2*-mutant tumors (NCT03284385). Given preclinical studies showing crosstalk between WEE1 and the mTOR/AKT/PI3K axis in regards to cell cycle regulation and cellular resource management [8, 9], further studies on sequential or combined PI3K and WEE1 inhibition and associated biomarker-development in *SETD2*-mutant ccRCC could be warranted.

While revolutionary treatment advances have been made in the past decade for patients with ccRCC, metastatic disease remains lethal and approved biomarker-driven strategies to guide treatment selection and order are lacking [4]. This current article adds to mounting preclinical evidence that the specific genomic background of a patient's ccRCC can drive cancer survival and progression, but also engender specific vulnerabilities that can be targeted. Additional preclinical and clinical studies on synthetic lethal treatment options will bring precision medicine more to the forefront for patients with ccRCC.
